# Exposure to ionizing radiations can cause hazardous effects on differentiation of human CD34+ hematopoietic stem cells

**DOI:** 10.1186/1755-8166-7-S1-P128

**Published:** 2014-01-21

**Authors:** Angshuman Biswas, Asiti Sarma, Subrata Kumar Dey

**Affiliations:** 1 Stem Cell Research Laboratory, West Bengal University of Technology, Kolkata – 700 064, India; 2 Radiation Biology Lab, Inter University Accelerator Centre, New Delhi- 110 067, India

## Statement of purpose

The objective of this study is to investigate effects of ionizing radiation on human hematopoietic stem cell differentiation.

## Background

Despite being a strong mutagen, causing several genetic and chromosomal aberrations, ionizing radiation in the form of gamma rays and heavy ion radiation is becoming increasingly important in medical therapies and cancer. Heavy ions especially the particle beam of carbon ion are high energy radiations that is considered to be extremely hazardous during occupational radiological emergencies, manned space missions, high altitude flights, accidents casualties etc. Healthy blood cells in human body arise by the differentiation of a very small population of pluripotent hematopoietic stem cells that have the capacity of self renewal throughout their lifespan. Effects of radiation whatsoever minimum on these unique cells thus have greater impact on the human system in both long term and short term scenario which might currently lead to an idea of a new concept leading to cancer stem cells.

## Methods

Umbilical cord blood collected in-utero was subjected to immuno-magnetic enrichment, followed by flowcytometric estimation for stem cell content. Isolated hematopoietic stem cells were then cultured into specialized cytokine based medium and exposed to various doses of ionizing radiation for investigations on CD34+ marker based flowcytometric survival assay, differentiation & clonogenic potential by CFC assay, genotoxicity and cytotoxicity.

## Results and conclusion

Exposure to different doses of ionizing radiation showed a marked difference in the survival of CD34+ stem cells in both dose and time dependant manner. Differentiation of stem cells into their adult progenitor cells were also altered with varying doses of radiation treatment. Our findings show variations on response of the human CD34+ stem cells when exposed to ionizing radiation which is significantly comparable to normal blood lymphocytes and cancerous K562 cells.

